# Personalized Language Training and Bi-Hemispheric tDCS Improve Language Connectivity in Chronic Aphasia: A fMRI Case Study

**DOI:** 10.3390/jpm15080352

**Published:** 2025-08-03

**Authors:** Sandra Carvalho, Augusto J. Mendes, José Miguel Soares, Adriana Sampaio, Jorge Leite

**Affiliations:** 1 Psychological Neuroscience Laboratory, Centro de Investigação em Psicologia (CIPsi), Department of Basic Psychology, School of Psychology, University of Minho, 4710-057 Braga, Portugal; 2 Laboratory of Neuroimaging of Aging (LANVIE), University of Geneva, 1205 Geneva, Switzerland; 3 Geneva Memory Center, Department of Rehabilitation and Geriatrics, Geneva University Hospitals, 1205 Geneva, Switzerland; 4 Life and Health Sciences Research Institute (ICVS), School of Medicine, University of Minho, 4710-057 Braga, Portugal; 5 RISE-Health, CINTESIS.UPT, Portucalense University, Rua Dr. António Bernardino de Almeida, 541, 4200-072 Porto, Portugal; jorgel@upt.pt

**Keywords:** non-fluent aphasia, transcranial direct current stimulation, language training, functional connectivity, cognitive training, personalized therapy

## Abstract

**Background**: Transcranial direct current stimulation (tDCS) has emerged as a promising neuromodulatory tool for language rehabilitation in chronic aphasia. However, the effects of bi-hemispheric, multisite stimulation remain largely unexplored, especially in people with chronic and treatment-resistant language impairments. The goal of this study is to look at the effects on behavior and brain activity of an individualized language training program that combines bi-hemispheric multisite anodal tDCS with personalized language training for Albert, a patient with long-standing, treatment-resistant non-fluent aphasia. **Methods**: Albert, a right-handed retired physician, had transcortical motor aphasia (TCMA) subsequent to a left-hemispheric ischemic stroke occurring more than six years before the operation. Even after years of traditional treatment, his expressive and receptive language deficits remained severe and persistent despite multiple rounds of traditional therapy. He had 15 sessions of bi-hemispheric multisite anodal tDCS aimed at bilateral dorsal language streams, administered simultaneously with language training customized to address his particular phonological and syntactic deficiencies. Psycholinguistic evaluations were performed at baseline, immediately following the intervention, and at 1, 2, 3, and 6 months post-intervention. Resting-state fMRI was conducted at baseline and following the intervention to evaluate alterations in functional connectivity (FC). **Results**: We noted statistically significant enhancements in auditory sentence comprehension and oral reading, particularly at the 1- and 3-month follow-ups. Neuroimaging showed decreased functional connectivity (FC) in the left inferior frontal and precentral regions (dorsal stream) and in maladaptive right superior temporal regions, alongside increased FC in left superior temporal areas (ventral stream). This pattern suggests that language networks may be reorganizing in a more efficient way. There was no significant improvement in phonological processing, which may indicate reduced connectivity in the left inferior frontal areas. **Conclusions**: This case underscores the potential of combining individualized, network-targeted language training with bi-hemispheric multisite tDCS to enhance recovery in chronic, treatment-resistant aphasia. The convergence of behavioral gains and neuroplasticity highlights the importance of precision neuromodulation approaches. However, findings are preliminary and warrant further validation through controlled studies to establish broader efficacy and sustainability of outcomes.

## 1. Introduction

Aphasia, predominantly caused by left-hemispheric stroke, is a debilitating language impairment that hinders speech production and/or understanding [1,2]. Non-fluent aphasia is generally characterized by impaired articulation, phonological processing, and syntactic deficits and is typically linked to lesions in anterior language regions, notably the inferior frontal gyrus (Broca’s area) [3]. Although spontaneous recovery may occur in the acute and subacute phases, most individuals reach a recovery plateau within the first year post-stroke [4,5]. During the chronic phase, aphasia frequently becomes resistant to standard therapy, resulting in several patients experiencing enduring communication impairments and few avenues for substantial recovery. This clinical stalemate highlights the pressing necessity for personalized, circuit-oriented therapies that might reactivate inactive brain networks. One promising approach for these circuit-oriented therapies involves transcranial direct current stimulation (tDCS), a non-invasive brain stimulation (NIBS) technique capable of modulating cortical excitability and promoting long-term plastic changes [6]. Conventional tDCS studies have demonstrated considerable success in targeting perilesional areas with anodal stimulation [7,8]; however, effect sizes are constrained and exhibit significant variability among patients. Recent meta-analyses underscore this diversity, identifying patient-specific characteristics such as lesion location, chronicity, and neuroanatomical variations as modifiers of response. Moreover, recent evidence-based guidelines emphasize that the overall level of evidence for tDCS in aphasia rehabilitation remains modest and heterogeneous, underscoring the need for rigorous, personalized interventions to clarify its clinical utility [9]. These results have led to a transition towards multisite and network-targeted methodologies, which seek to influence scattered cortical networks associated with language processing by synchronizing stimulation targets with neurocognitive models [10,11]. From a cognitive neuroscience perspective, recovering language after a stroke depends on keeping a healthy balance of excitation and inhibition across both hemispheres. Classic and newer models point out that, when the right hemisphere is too active, it can actually slow down the recovery of left-hemisphere language skills [5]. Notably, some studies suggest that tDCS may help adjust this balance, but its effects can differ depending on how and where it is applied [7,8,12]. For example, there is growing research into using bi-hemispheric or multisite tDCS to boost activity in damaged areas while dialing down overactivity in the opposite hemisphere [8,10]. Building on this, we believed that combining bi-hemispheric tDCS with tailored language training could better support the brain’s own reorganization process and help people regain both speech and understanding. In our previous study, we demonstrated that multisite tDCS targeting the dorsal stream of language, combined with adaptive cognitive training, produced significant short- and medium-term improvements in speech production in a person with chronic non-fluent aphasia [13]. These behavioral gains were accompanied by changes in functional connectivity in left-lateralized language networks, supporting the relevance of network-guided neuromodulation approaches. The language training in that study was tailored to address the individual’s specific weaknesses in phonological, lexical, and syntactic areas. This method changes the difficulty and content of each session based on how the patient’s language skills are changing, which is different from normal rehabilitation protocols that usually employ predefined task sets.

This study builds upon previous research by investigating a bi-hemispheric multisite anodal tDCS montage in a second example of chronic non-fluent aphasia, emphasizing bilateral dorsal stream activation. This study builds upon our prior unilateral approach by investigating the feasibility and effects of a bi-hemispheric multisite montage combined with language-focused training in a single-case design. We posited that this integrated intervention would facilitate the recovery of both speech production and understanding by augmenting functional restructuring within and across hemispheres. This is one of the first case studies to systematically combine bi-hemispheric, network-targeted tDCS with individualized language instruction in a patient with chronic, treatment-resistant aphasia. Even with these encouraging developments, the effects of bi-hemispheric multisite tDCS on behavioral and neurological outcomes in chronic aphasia are still insufficiently investigated.

## 2. Materials and Methods

### 2.1. Ethical Approval

The study was conducted in accordance with the Declaration of Helsinki and approved by the Ethics Committee for Life and Health Sciences (Comissão de Ética para as Ciências da Vida e da Saúde) of the University of Minho (protocol number SECVS 014/2016, approval date: 17 June 2016). Written informed consent was obtained from the participant for both participation and publication of anonymized clinical data and images.

### 2.2. Case Presentation

**Figure 1:**
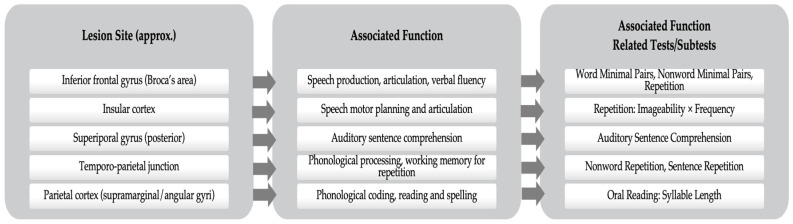
Approximate lesion–function associations relevant to dorsal language stream targeting in chronic non-fluent aphasia.

This case study followed a protocol similar to the one previously published by our group [13], which combined adaptive language training with multisite tDCS in chronic post-stroke aphasia. The participant was a right-handed adult male who, at the age of 55, experienced an ischemic stroke in the left middle cerebral artery shortly after surgery for a carotid body tumor. In the acute phase, he was diagnosed with TCMA, which typically involves lesions in the left frontal lobe, especially the supplementary motor area (SMA) just anterior and superior to Broca’s area, leading to profound speech production difficulties. Consistent with this, neuroimaging in the chronic stage revealed generalized cortical atrophy in the left hemisphere, with widening in fronto-temporo-parieto-insular regions. This lesion pattern likely affects multiple language-related areas, including the inferior frontal gyrus (Broca’s area), insular cortex, superior temporal gyrus, temporo-parietal junction, and supramarginal/angular gyri. These areas are known to support functions such as speech production, articulation, phonological processing, auditory comprehension, and reading and spelling. Clinically, this is reflected in persistent articulation and fluency deficits, with output limited to short phrases or single-word utterances. The specific links between lesion sites, functional domains, and the psycholinguistic tasks used are summarized in Figure 1; these lesion–function relationships were informed by well-established neurocognitive models of language networks and dorsal stream processing [14,15].

Neuropsychological assessments also revealed impairments in attention, working memory, and executive functioning. Although he initially exhibited right-sided hemiparesis, full motor recovery was achieved by the time of this intervention.

Over the following years, Albert demonstrated persistent deficits in verbal fluency and speech articulation. His expressive language remained limited to short, fragmented utterances composed primarily of basic nouns and verbs. He experienced substantial difficulty conveying complex ideas and understanding syntactically demanding or lengthy sentences. Despite undergoing multiple rounds of conventional speech-language therapy (SLT) and participating in online cognitive rehabilitation programs, these efforts resulted in only modest, short-lived improvements. Consequently, Albert continued to face significant challenges in everyday communication and social interaction.

At baseline, Albert presented marked deficits across various language domains, including verbal working memory, phonemic and semantic fluency, auditory sentence processing, reading, spelling, and lexical retrieval. In contrast, his non-verbal cognitive abilities—such as visuospatial construction, visual working memory, attention, and processing speed—were preserved. He reported no clinically significant symptoms of depression or anxiety. Notably, he had no prior exposure to NIBS, including tDCS.

Given the chronicity and severity of Albert’s language impairment, a tailored intervention was developed to target both expressive and receptive language functions. The treatment protocol combined a personalized language training program with a bi-hemispheric, multisite anodal tDCS montage targeting bilateral dorsal language streams.

Conventional interhemispheric rebalancing methods generally employ anodal stimulation on the left hemisphere and cathodal on the right to mitigate maladaptive activity in the right hemisphere, as demonstrated by You et al. [12]. However, recent findings indicate that bilateral anodal stimulation with extracephalic references can enhance network-level plasticity without directly suppressing right-hemispheric areas [9,10]. This method may promote collaborative reconfiguration across both hemispheres, particularly when stimulation is administered simultaneously with functionally focused training [7,8].

In our approach, anodes were positioned over the bilateral dorsal stream regions (F5/CP5 on the left and F6/CP6 on the right), while extracephalic cathodes on the shoulders mitigated direct inhibitory effects on cortical areas, thereby enhancing the focused regulation of language networks. This dispersed, excitatory arrangement signifies a transition from therapies focusing on focal lesions to those influenced by network dynamics, prioritizing functional connection and collaboration above mere suppression.

### 2.3. Study Design and Timeline

This open-label case study comprised a baseline assessment, an intervention phase combining adaptive language training with multisite tDCS, and a series of follow-up assessments extending up to six months post-intervention. Resting-state functional MRI (rs-fMRI) scans were acquired at baseline and immediately after the final session to assess changes in functional connectivity. EPI images were not included in the figure due to their lower spatial resolution and T2-contrast.

Follow-up assessments were conducted at five timepoints: immediately post-intervention and at 1, 2, 3, and 6 months. To reinforce treatment effects, a booster session replicating the original intervention protocol was administered on the same day as each follow-up assessment. The intervention specifically targeted the dorsal language stream, with the goal of enhancing speech production by increasing activity in left perilesional areas through multisite anodal tDCS.

### 2.4. Assessments and Follow-Ups

The baseline and follow-up assessments focused exclusively on psycholinguistic testing using the Portuguese adaptation of the Psycholinguistic Assessments of Language Processing in Aphasia (PALPA-P) [16]. Language production was evaluated through the following subtests: Word Minimal Pairs, Nonword Minimal Pairs, and Repetition (Imageability × Frequency, Nonword, and Sentence). Language comprehension was assessed using the auditory sentence comprehension and locative relations subtests. Reading abilities were evaluated via the Spoken Letter–Written Letter Matching and Syllable Length tasks. Clinical scales were not administered due to Albert’s difficulty in completing self-report questionnaires.

#### Statistical Analysis

To evaluate whether changes in Albert’s performance were statistically and clinically meaningful, we calculated the Reliable Change Index (RCI) for each relevant subtest of the PALPA-P across follow-up assessments. The RCI is a well-established method used to determine whether the difference between pre- and post-intervention scores exceeds what would be expected due to measurement error or natural variability [17].

RCI values were computed using the following formula:

$$RCI=\frac{Xpost-Xpre}{SEdiff}$$

where SE_diff = √2 × SEM and SEM (standard error of measurement) were derived from normative data validated for the Portuguese version of the PALPA [16]. An RCI value greater than ±1.96 was considered statistically significant at the 95% confidence level (*p* < 0.05; two-tailed). This method ensured that the changes observed across follow-up timepoints reflected true, reliable improvements rather than measurement error or random fluctuations.

### 2.5. Language Training

The language training program adhered to the same technique as utilized in the prior case of Mary [13] but was tailored to Albert’s specific impairments found at the baseline PALPA-P assessment. Tasks were chosen and arranged to address critical deficits in phonological processing, syntactic comprehension, and lexical retrieval. This adaptive method sought to enhance language rehabilitation by aligning the cognitive demands and linguistic intricacy of each assignment with Albert’s developing abilities, thereby promoting engagement and neuroplasticity during the intervention. The intervention consisted of 15 language training sessions, with each session lasting two hours, three times per week (totaling 30 h). Additional booster sessions were administered on the same day as each follow-up psycholinguistic assessment (except at the 1-year follow-up), bringing the total number of sessions to 20. Training in speech production focused on phonological processing and repetition tasks (see Table 1). Stimuli were selected from the European Portuguese lexical database Minho Word Pool [18] and controlled for frequency and imageability. Words were distributed across sessions in order of difficulty, ranging from high-frequency/high-imageability items (e.g., água [water]) to low-frequency/low-imageability items (e.g., reversível [reversible]). None of the trained items were used in the assessments or follow-ups. Exercises included phoneme identification, deletion, replacement, and discrimination. Repetition exercises used both the lexical stimuli and specially constructed pseudowords. For speech comprehension, tasks required the participant to answer questions involving complex sentence structures and to process locative relationships. This dual focus aimed to simultaneously target both production and comprehension deficits identified at baseline.

### 2.6. tDCS Parameters

Bi-hemispheric, multisite transcranial direct current stimulation (tDCS) was administered using two independent stimulators: the HDCStim (Newronika, Milan, Italy) and the Eldith DC Stimulator Plus (NeuroConn, Ilmenau, Germany). The bi-hemispheric, multisite approach was guided by the principle of interhemispheric rebalancing, aiming to upregulate activity in the lesioned (left) hemisphere while downregulating maladaptive overactivation in the contralesional (right) hemisphere. The goal of stimulating both anterior and posterior language areas bilaterally was to promote coordinated plasticity changes throughout the distributed language networks. Each device delivered stimulation to one hemisphere, targeting language-relevant regions corresponding to Broca’s and Wernicke’s areas. Stimulation was delivered via four saline-soaked anodal electrodes (25 cm^2^; 5 × 5 cm), positioned over F5, F6, CP5, and CP6 according to the 10–20 EEG system. This bi-hemispheric, multisite setup was developed to reach both sides of the brain’s dorsal language pathways, following the idea of rebalancing how the two hemispheres work together. F5 and F6 approximate the lateral frontal areas involved in speech production and phonological processing (including Broca’s area and its right-hemisphere homolog), while CP5 and CP6 approximate posterior parietal regions such as the supramarginal gyrus and temporo-parietal junction, which support phonological working memory and repetition. By placing the anodes over F5/CP5 on the damaged (left) side and F6/CP6 on the opposite (right) side—and using extracephalic cathodes—the goal was to boost activity in areas near the lesion while also helping to calm any overactivity on the right. This approach is supported by modeling work showing that giving anodal stimulation to both hemispheres with this setup can strengthen network-level plasticity without directly suppressing the right hemisphere, which may help the whole language system reorganize more effectively [10,11]. Two large extracephalic reference electrodes (100 cm^2^; 10 × 10 cm) were placed over the ipsilateral shoulders to minimize cortical current flow beneath the cathodes. A current of 1 mA was applied to each anode (current density = 0.04 mA/cm^2^) for 20 min, with 15 s ramp-up and ramp-down periods. Stimulation was applied concurrently with the language-focused colanguages training tasks. This timing was chosen to promote activity-dependent plasticity by enhancing cortical excitability while the participant was actively engaging language networks related to phonological processing, syntactic comprehension, and lexical access (Figure 1). Throughout all sessions, Albert was continuously monitored for adverse effects such as skin irritation, headache, dizziness, or discomfort at the electrode sites. No adverse events were reported, and tolerability was rated as high across all sessions. Impedance levels were checked prior to each session to ensure they remained below manufacturer-recommended thresholds.

### 2.7. Resting-State fMRI

#### 2.7.1. Data Acquisition

Resting-state functional MRI (rs-fMRI) data were collected using a Siemens Magnetom Tim Trio 3.0 T MRI system (Siemens Medical Solutions, Erlangen, Germany). A 7 min acquisition was performed, yielding 210 volumes using a BOLD-sensitive echo-planar imaging (EPI) sequence with the following parameters: repetition time (TR) = 2000 ms; echo time (TE) = 29 ms; flip angle = 90°; field of view (FOV) = 1554 mm; matrix size = 64 × 64; in-plane resolution = 3 × 3 mm^2^; and slice thickness = 3 mm across 39 contiguous axial slices. During the scan, the participant was instructed to remain awake with eyes closed and to refrain from engaging in any specific mental task. Self-report after scanning confirmed compliance and absence of sleep.

#### 2.7.2. Preprocessing of fMRI Data

Visual inspection of the raw data ensured the absence of significant motion artifacts. To allow for signal equilibrium and participant adaptation to scanner noise, the first five volumes were excluded from further analysis. Preprocessing was carried out using the SPM12 software package R2023b (9.15) (Statistical Parametric Mapping; Wellcome Trust Centre for Neuroimaging, London, UK). Slice-timing correction was applied using the first slice as a temporal reference and Fourier phase shift interpolation. Realignment was performed using a six-parameter rigid-body transformation, with realignment parameters estimated at 0.9 quality, a 4 mm sampling distance, and 5 mm FWHM Gaussian kernel smoothing. Only scans with a translational displacement < 2 mm and a rotational movement < 1° were retained.

Spatial normalization to the MNI standard space was performed via non-linear registration to the EPI template using trilinear interpolation. The normalized data were subsequently smoothed with an 8 mm FWHM Gaussian kernel to reduce spatial noise. Temporal filtering was conducted using a band-pass filter (0.01–0.08 Hz), and linear trends were removed to eliminate low-frequency drifts and high-frequency noise.

#### 2.7.3. Independent Component Analysis and Network Identification

Independent component analysis (ICA) was implemented using the Group ICA of fMRI Toolbox (GIFT v4.0b; [19]; https://sourceforge.net/projects/icatb/). The pipeline involved three primary stages: dimensionality reduction via Principal Component Analysis (PCA), estimation of group-level independent components, and subject-specific back-reconstruction. A total of 20 independent components were extracted based on an optimal trade-off between spatial specificity and statistical reliability, using the Infomax algorithm. To assess the stability of component estimation, the ICASSO framework was employed with 20 repetitions.

The resulting components, expressed as t-statistic maps, were transformed into Z-score maps to reflect the relative contribution of individual voxels to each component. These components were visually examined and spatially correlated with canonical resting-state networks (RSNs) based on established templates [20]. To evaluate connectivity changes, Z-score maps from baseline and post-intervention were contrasted. Differences were considered significant when exceeding a Z-score threshold of ±2.5 and a minimum cluster extent of 10 voxels, for the independent components of each moment (i.e., baseline and post-intervention maps analyzed separately prior to direct comparison). This thresholding ensured that only spatially meaningful activations were retained before contrast analysis. The final contrast maps between timepoints could therefore display voxel-wise Z-values lower than 2.5, as only thresholded component maps entered this step.

## 3. Results

### 3.1. Neuropsychological Assessment

**Figure 2:**
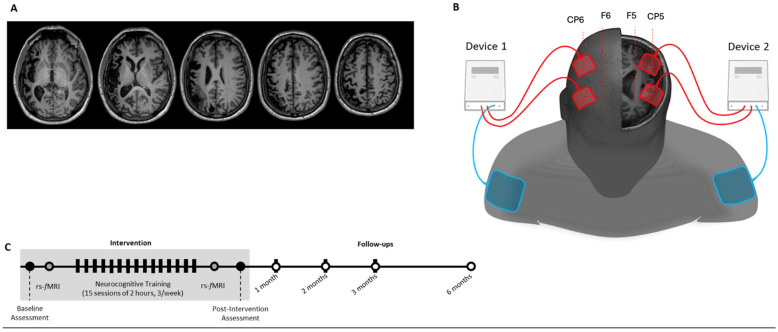
(**A**) Lesion localization visualized through structural MRI. (**B**) Setup for bi-hemispheric multisite anodal tDCS using two independent stimulators. Anodes were placed over the left hemisphere (F5, CP5; lesioned side) and right hemisphere (F6, CP6; contralesional side) according to the 10–20 EEG system, with large extracephalic reference electrodes positioned on the ipsilateral shoulders to minimize unwanted cortical current flow beneath the cathodes. (**C**) Timeline of the intervention, showing 15 sessions of personalized language training combined with tDCS, resting-state fMRI scans at baseline and post-intervention, and follow-up assessments at 1, 2, 3, and 6 months.

Albert did not show significant improvements in phonological processing (*p* > 0.05), while significant effects were observed in sentence comprehension and reading and spelling of PALPA-P (Table 1) in several follow-ups (i.e., post-intervention and at 1 month, 2 months, 3 months, and 6 months) when compared with baseline assessment. In sentence comprehension, a statistical improvement was found in auditory sentence comprehension (1 month: RCI = 4.97, *p* < 0.001; 2 months: RCI = 5.42, *p* < 0.001; and 3 months: RCI = 4.51, *p* < 0.001). Moreover, in reading and spelling, a statistical improvement was observed in oral reading: syllable length (1 month: RCI = 1.96, *p* = 0.025) (Figure 2). Every other comparison was not statistically significant (*p* > 0.05) (Table 1).

**Figure 3:**
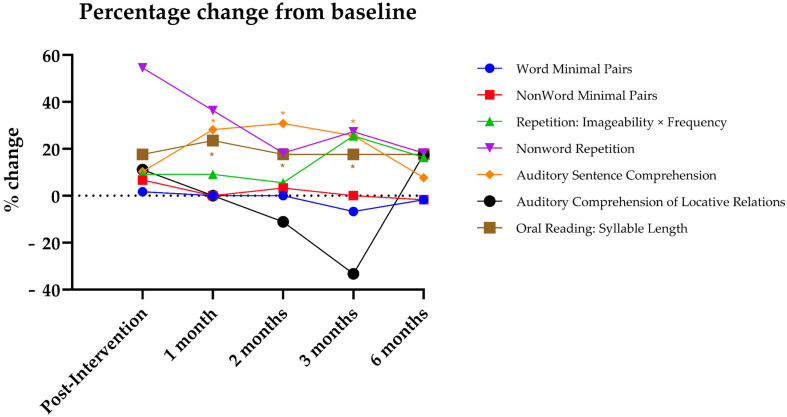
Percentage change from baseline across psycholinguistic subtests at each timepoint.

In percentage change from baseline, descriptive trends demonstrated varied patterns over the psycholinguistic subtests (Figure 3). Phonological discrimination tasks (Word Minimal Pairs and Nonword Minimal Pairs) demonstrated little to no change, with performance at or close to baseline or slightly below at later follow-ups. In contrast, Repetition, Imageability × Frequency, exhibited a steady increase, peaking at approximately 25% improvement at three months. Nonword Repetition showed the most significant immediate gain following intervention (over 50%), although this declined somewhat with duration, but remained above baseline. Sentence Repetition was minimal at all periods. Auditory sentence comprehension showed the most significant and long-lasting improvement, with a percentage gain of over 30% at two months, and remaining above baseline at six months. Oral reading, Syllable Length, also showed a consistent improvement of around 17% at all follow-up periods. These percentage values are provided only for descriptive purposes and complement the RCI analysis that has been performed using raw scores.

### 3.2. Resting-State fMRI Results

**Figure 4:**
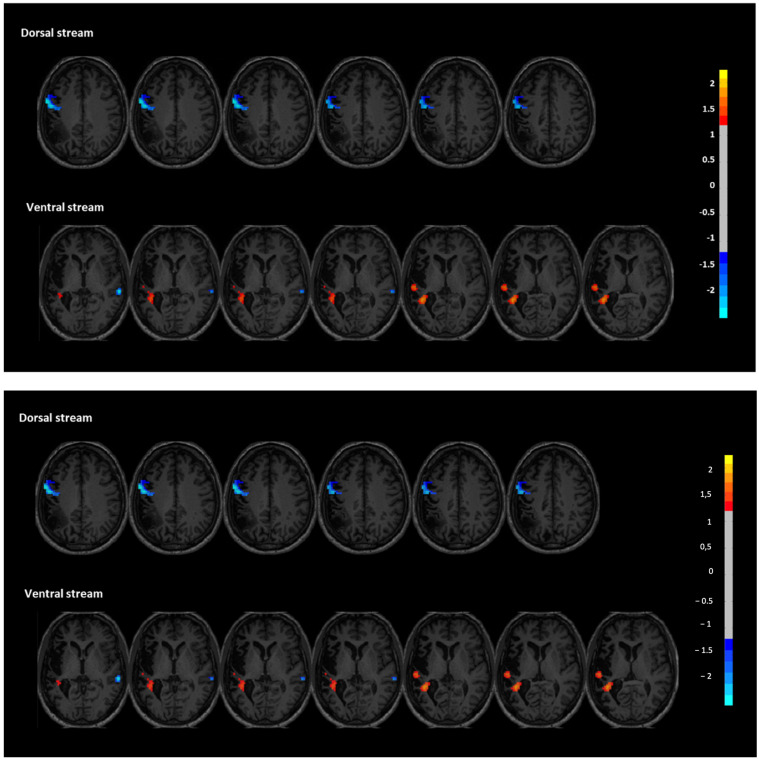
Functional connectivity changes in dorsal (**top**) and ventral (**bottom**) language streams after the intervention. Z-score maps show increased connectivity in left temporal areas (red) and decreased connectivity in right-hemispheric regions (blue). Color bar indicates voxel-wise Z-score differences between post- and pre-intervention resting-state fMRI.

Functional connectivity (FC) analyses revealed distinct post-intervention changes in both the dorsal and ventral language streams, supporting a reorganization of language-related networks. In the dorsal stream, FC was significantly decreased in the left inferior frontal and precentral regions (peak coordinates: X = −60; Y = 0; and Z = 33), with a cluster size of 139 voxels and a z-score of −0.894. Note the following: These Z-scores represent voxel-wise differences between timepoints and may be lower than the initial ±2.5 threshold applied to the independent components, as explained in the Methods. This reduction may reflect a normalization or rebalancing of hyperconnectivity in perilesional motor-speech regions often observed in chronic aphasia. No significant increases were found in the dorsal regions of the right hemisphere. In contrast, the ventral stream showed signs of enhanced engagement. Specifically, two clusters within the left superior temporal gyrus demonstrated increased FC (X = −33; Y = −42; and Z = 12 and X = −54; Y = −24; and Z = 6), with cluster sizes of 109 and 12 voxels, and z-scores of 1.223 and 0.934, respectively. These changes suggest a strengthened role of left temporal areas, which are critical for auditory comprehension and phonological decoding. Additionally, decreased FC was observed in the right superior temporal region (X = −42; Y = 15; Z = −12), with a cluster size of 12 and a z-score of −0.806. This finding is consistent with the hypothesis that reducing maladaptive right-hemisphere overactivation may facilitate improved language function through interhemispheric rebalancing. Together, these findings suggest that the intervention facilitated plastic changes that increased connectivity of the left-hemispheric language network while downregulating compensatory activity in the right hemisphere, particularly in the ventral stream (Figure 4).

## 4. Discussion

This case report shows the effectiveness of a precision-guided neuromodulatory intervention in a patient with chronic, non-fluent aphasia that had been resistant to multiple prior treatments. Albert had undergone several sessions of conventional speech-language therapy and cognitive rehabilitation without achieving lasting functional enhancements. His persistent expressive and receptive language deficits—nearly a decade post-stroke—underscore the therapeutic challenges inherent in chronic aphasia and the pressing need for customized, circuit-level interventions.

This single-case study expands upon our prior research [13] by investigating the effects of bi-hemispheric multisite anodal tDCS in conjunction with language-focused training in an individual with chronic non-fluent aphasia. Our previous research showed that unilateral multisite tDCS targeting the dorsal stream could enhance behavioral and neurological aspects of speech production and comprehension. The present study examines the supplementary effects of a bi-hemispheric montage.

**Figure 5:**
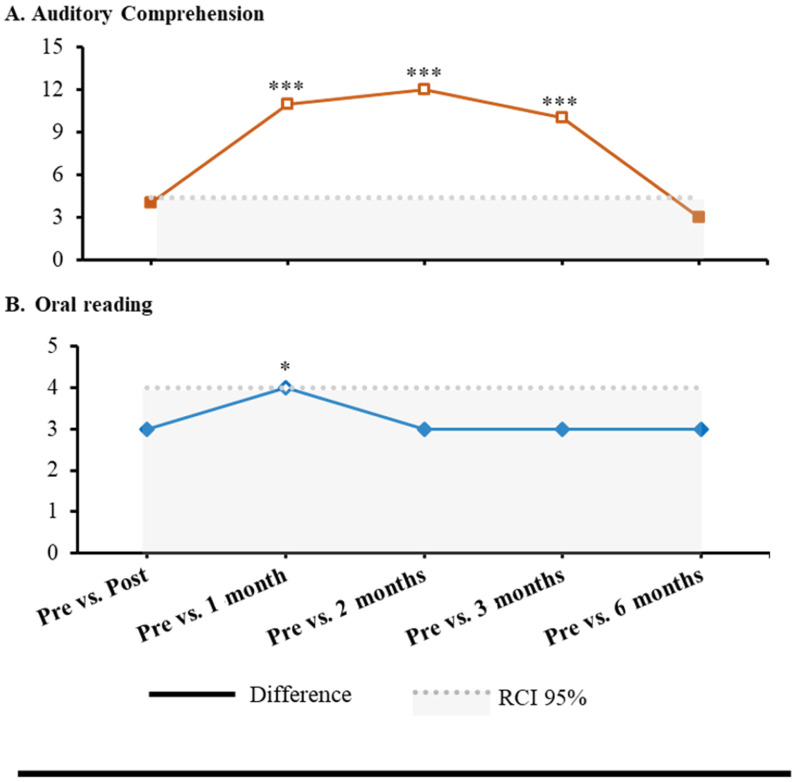
Improvements in auditory comprehension (**A**) and oral reading (**B**) following multisite tDCS. Significant differences are indicated by asterisks. The line represents change from baseline; the shaded area indicates the 95% Reliable Change Index (RCI) threshold. * *p* < 0.05; *** *p* < 0.001.

By stimulating both hemispheres simultaneously and assessing outcomes through repeated behavioral testing and resting-state functional connectivity (rs-fMRI), this study extends prior findings of the usefulness of the combination between tDCS and language training and provides further insight into the interhemispheric mechanisms of recovery in a participant with aphasia. We hypothesized that this bilateral approach would facilitate functional restructuring across hemispheres and augment improvements in both expressive and receptive language functions. Our results provided partial validation for this idea. Albert showed statistically significant improvements in auditory sentence comprehension and oral reading, particularly at the 1- and 3-month follow-ups. These RCI-based results were further supported by the descriptive percentage-change trends (Figure 5), which showed the strongest sustained gains in auditory sentence comprehension and oral reading, while phonological discrimination tasks remained largely unchanged. These behavioral improvements were accompanied by enhanced FC in left-lateralized language networks, alongside diminished connection in right-hemispheric areas normally linked to maladaptive plasticity in aphasia. We chose to use this bi-hemispheric multisite setup because newer network-based research suggests that stimulating both hemispheres can help the brain reorganize language networks in a more balanced way, rather than just shutting down the overactive right side [10,11]. By placing the anodes over F5/CP5 on the left (lesioned) hemisphere and F6/CP6 on the right (contralesional) side, and using extracephalic cathodes, our aim was to boost activity near the damaged areas while also calming down any unhelpful overactivity on the right. This strategy draws on modeling studies showing that bilateral anodal stimulation with extracephalic references can promote broader network plasticity without directly suppressing the right hemisphere—which may be especially helpful when someone still has severe, long-standing language difficulties.

The improvements in phrase comprehension noted in Albert’s instance are likely due to the downregulation of activity in the right superior temporal gyrus, along with enhanced connection in the left superior temporal lobe. The observed modifications in the brain align with previous studies suggesting that the modulation of right-hemisphere hyperactivity—through direct suppression or enhanced activation of left-hemisphere language regions—can lead to improved linguistic outcomes [12,21]. The absence of notable improvements in phonological processing corresponds with the diminished functional connectivity noted in the left inferior frontal and precentral regions, which are essential for phonological encoding and articulatory planning. This reduced connectivity could be a normalization of hyperconnectivity normally observed in perilesional regions in chronic aphasia. However, it is noteworthy that this network-level adjustment did not accompany clear improvements in phonological production. One possible explanation is that one of the causes of this lack of improvement is that the bilateral montage potentially spreads out the excitatory input too diffusely, rather than concentrating it onto the left inferior frontal areas involved in phonological encoding. This underscores the importance of paying extra care in designing the stimulation montage when one has to activate specific language functions.

This pattern is different from our previous case study that used unilateral multisite anodal tDCS, which showed that connectivity in left perilesional regions increased and phonological tasks improved as a result [13]. The results from Albert’s case indicate that bi-hemispheric anodal stimulation might elicit distinctive neuronal dynamics, potentially by interrupting or spreading concentrated activity in the left hemisphere within chronically compromised networks. This may elucidate why, in this instance, we noted more uniform improvements in sentence comprehension and oral reading, which depend more significantly on temporal and ventral stream processing, where enhanced functional connectivity was observed. The case-specific variations highlight the significance of stimulation montage and target network selection, emphasizing the necessity for personalized, network-informed strategies in neuromodulation therapy for aphasia. Moreover, this divergence in outcomes highlights the need for further investigation into how unilateral versus bilateral stimulation strategies may differentially impact distinct language components, such as phonology versus comprehension, particularly in chronic and treatment-resistant profiles.

Our results support the idea that using tDCS with language training in a network-specific and functionally directed way can improve recovery by activating specific brain circuits. Specifically, the execution of personalized cognitive training may have contributed to behavioral and neurological enhancements. Aligning each task with Albert’s unique language profile probably made him more interested in the tasks and activated the dorsal and ventral language streams in a way that was relevant to the tasks. This tailored integration of targeted cognitive deficit training with individualized tDCS montages enhances the progression of precision-guided neurorehabilitation.

It is worth noting that recent systematic reviews and meta-analyses continue to stress both the promise and the limitations of tDCS in language rehabilitation. For instance, Godoi et al. recently reviewed studies in primary progressive aphasia (PPA) and found modest yet significant improvements in general naming and spelling, though they did not see clear benefits for broader language domains such as comprehension or working memory [21]. Even though PPA is etiologically different from post-stroke aphasia, these findings resonate with our own, suggesting that tDCS effects are likely to be domain-specific and influenced by how the stimulation is applied, from montage design to the choice of network targets. In our case study, the bi-hemispheric tDCS combined with individualized training seemed to boost sentence comprehension and oral reading but did not significantly impact phonological skills. This highlights how important it is to clarify which aspects of language are most sensitive to specific tDCS configurations and how tailored cognitive exercises may amplify these gains.

Interestingly, these observations also match what is being found in other neurodegenerative language disorders. Lomi et al., for example, conducted a meta-analysis that showed noninvasive brain stimulation (NiBS), whether tDCS or TMS, applied alone or alongside standard speech therapy, did not yield major benefits compared to sham in most language tasks for PPA patients, although there was a slight trend favoring combined approaches [22]. Overall, these results underline the importance of customizing both the stimulation protocol and the language intervention to achieve meaningful clinical outcomes.

These insights are consistent with our finding that the biggest improvements happened in the first three months, but booster sessions at each follow-up may have helped sustain the effects for a short time. The absence of substantial improvement after six months underscores the urgent necessity for more rigorous or continuous reinforcement tactics, such as repeated booster cycles or adaptive dose schedules, to extend the therapeutic effects. Building on these findings, integrating EEG-derived biomarkers and personalized tACS protocols—as recently explored by Mendes et al. for tailoring tACS to individual P3 oscillations—could offer an adaptive, closed-loop approach to sustain neuroplastic changes more effectively and tailor stimulation to each patient’s individual oscillatory patterns [23,24]. These results are consistent with prior studies demonstrating that therapy persistence remains a major challenge in post-stroke aphasia rehabilitation. In neurorehabilitation, it is quite common to include booster sessions because they help patients hold on to their progress or even build on it over time. But these extra sessions also make it harder to tell exactly how much of the improvement comes from the main treatment and how much comes from the additional boost. This approach is consistent with principles of motor learning and evidence supporting distributed and intensive practice in aphasia rehabilitation [25,26,27]. Recent evidence also indicates that combining speech and language therapy with non-invasive brain stimulation or other adjuncts may lead to superior outcomes compared to standard therapy alone, further supporting the importance of well-structured, multimodal, and sustained interventions [28]. In the future, it would be worth comparing groups who do and do not get booster sessions to see how much they really help people maintain their language gains.

This case study offers useful insights into how personalized, network-guided neuromodulation might help people with chronic aphasia, but it also has important limitations. Because it is a single-case report without a sham condition, the findings should be seen as preliminary and cannot be generalized until they are tested in larger, controlled studies. We only used performance-based language assessments and could not include self-reported measures, like quality of life or confidence in communication, because of the participants’ severe language difficulties. This means we do not fully know how the patient felt about any real-life changes. Future studies should try to combine objective and subjective measures for a clearer picture. We also recognize that giving booster sessions on the same day as follow-up assessments was meant to help keep the effects going, but it may have boosted short-term improvements, making it harder to know what really lasted. Using the Reliable Change Index without adjusting for multiple comparisons is another limitation of this type of study. Finally, reporting effect size estimates in larger samples would help show how meaningful these changes truly are.

## 5. Conclusions

This case study gives encouraging evidence that very individualized, network-targeted neuromodulation can help patients with chronic, treatment-refractory non-fluent aphasia attain meaningful improvement. After nearly a decade of typical therapy, Albert still had severe expressive and receptive language deficits. By synergistically pairing bi-hemispheric multisite anodal tDCS with language training based on his personal needs, we observed changes that suggest it is feasible to re-activate language networks even decades following stroke.

Of most note, Albert showed consistent progress in sentence comprehension and reading aloud that lasted for three months, a reflection of the brain’s power to alter through neuroplasticity, even with chronic state. These gains were not sustained at six months, showing that stronger or ongoing follow-up intervention might be required in order to support continued progress with time. Neuroimaging evidence may be provided in favor of this behavioral trend, with greater lateralization in left-hemisphere language areas and less maladaptive activity on the right. The failure to achieve visible improvement in phonological processing is also a reminder that how we make up the stimulation montage matters—that is targeting specifically is essential when disparate components of language rely on different circuits.

As a whole, this study aligns with the idea that network-based, bilateral tDCS approaches can be a viable option where traditional therapy alone has been optimized. Subsequent studies must explore ways to optimize stimulation parameters and pair them with neurophysiological indicators like EEG in order to forecast who would be most likely to benefit. According to these results, the use of adaptive closed-loop tACS protocols based on real-time EEG could be an exciting next step in more personalized and long-term neurorehabilitation of patients with chronic aphasia.

## Figures and Tables

**Table 1:** Psycholinguistic and clinical assessment scores at baseline and across follow-up timepoints.

Psycholinguistic and Clinical Assessment Scores						
	Baseline	Post-Intervention	1-Month Follow-Up	2-Month Follow-Up	3-Month Follow-Up	6-Month Follow-Up
Auditory Processing						
Word Minimal Pairs (max. score = 64)	60	59	60	60	56	59
Nonword Minimal Pairs (max. score = 64)	60	56	60	62	60	59
Repetition: Imageability x Frequency (max. score = 160)	55	60	60	58	69	64
Nonword Repetition (max. score = 30)	11	17	15	13	14	13
Sentence Repetition (max. score = 36)	0	2	2	0	0	0
Sentence Comprehension						
Auditory Sentence Comprehension (max. score = 60)	39	43	50 *	51 *	49 *	42
Auditory Comprehension of Locative Relations (max. score = 24)	9	10	9	8	6	9
Reading and Spelling						
Oral Reading: Syllable Length (max. score = 24)	17	20	21 *	20	20	20

Note: Scores with asterisks represent significant difference in comparison with baseline (RCI > 1.96; *p* < 0.05).

## Data Availability

The data supporting the findings of this study are not publicly available due to privacy and ethical restrictions but may be made available upon reasonable request. Interested researchers may contact the corresponding author (sandrarc@psi.uminho.pt) to inquire about access, which is subject to institutional approval and data sharing agreements.

## Abbreviations

The following abbreviations are used in this manuscript:

tDCS	Transcranial Direct Current Stimulation
TCMA	Transcortical Motor Aphasia
CT	Cognitive Training
fMRI	Functional Magnetic Resonance Imaging
rs-fMRI	Resting-State Functional Magnetic Resonance Imaging
FC	Functional Connectivity
PALPA	Psycholinguistic Assessments of Language Processing in Aphasia
NIBS	Non-Invasive Brain Stimulation
SLT	Speech-Language Therapy
ROI	Region of Interest
MNI	Montreal Neurological Institute
ICA	Independent Component Analysis
PCA	Principal Component Analysis
RSN	Resting-State Network
RCI	Reliable Change Index
